# Changes in Channel Trafficking and Protein Stability Caused by LQT2 Mutations in the PAS Domain of the HERG Channel

**DOI:** 10.1371/journal.pone.0032654

**Published:** 2012-03-02

**Authors:** Carol A. Harley, Catarina S. H. Jesus, Ricardo Carvalho, Rui M. M. Brito, João H. Morais-Cabral

**Affiliations:** 1 IBMC, Instituto de Biologia Molecular e Celular, Universidade do Porto, Porto, Portugal; 2 Centre for Neuroscience and Cell Biology, University of Coimbra, Coimbra Portugal; 3 Chemistry Department, Faculty of Science and Technology, University of Coimbra, Coimbra Portugal; Virginia Commonwealth University, United States of America

## Abstract

Inherited human long-QT2 syndrome (LQTS) results from mutations in the gene encoding the HERG channel. Several LQT2-associated mutations have been mapped to the amino terminal cytoplasmic Per-Arnt-Sim (PAS) domain of the HERG1a channel subunit. Here we have characterized the trafficking properties of some LQT2-associated PAS domain mutants and analyzed rescue of the trafficking mutants by low temperature (27°C) or by the pore blocker drug E4031. We show that the LQT2-associated mutations in the PAS domain of the HERG channel display molecular properties that are distinct from the properties of LQT2-associated mutations in the trans-membrane region. Unlike the latter, many of the tested PAS domain LQT2-associated mutations do not result in trafficking deficiency of the channel. Moreover, the majority of the PAS domain mutations that cause trafficking deficiencies are not rescued by a pore blocking drug. We have also explored the *in vitro* folding stability properties of isolated mutant PAS domain proteins using a thermal unfolding fluorescence assay and a chemical unfolding assay.

## Introduction

The human *ether-a-go-go* (eag) related gene (HERG) [1]–[2] encodes the rapidly activating delayed rectifier K^+^ channel (IKr) [1], [3], an important component in the repolarization of the cardiac action potential. Mutations in HERG cause human long QT2 syndrome (LQT2) which is a heart condition in which delayed repolarization of the heart, characterized by a prolongation of the electrocardiogram QT interval, increases the risk of life threatening arrhythmias and sudden death [4], [5].

Native HERG channels are proposed to be hetero-tetramers arising from the assembly of 1a and 1b α-subunits encoded by alternate transcripts of the HERG gene [6], [7]. HERG 1a and 1b subunits have an identical core containing six transmembrane-spanning helices (S1–S6) and long conserved carboxyl terminal domains. HERG 1a and 1b subunits differ in the length of their amino terminus: ∼396 residues in the HERG 1a subunit and ∼56 residues in the HERG 1b subunit. The crystal structure of the first ∼135 residues in the HERG 1a subunit has been determined and has been shown to contain a conserved Per-Arnt-Sim (PAS) domain [8]. The functional role of the HERG PAS domain is not known but it is thought to participate in regulation of channel function. In other proteins PAS domains have been shown to sense environmental stimuli (light, ligands, and redox potentials) and regulate a variety of biochemical processes in both eukaryotic and prokaryotic systems [9]. In the HERG channel, removal of the entire PAS domain or the presence of LQT2-associated missense mutations in the PAS domain region of the HERG channel has been shown to accelerate the rate of deactivation of the channel [10], [11]. Previous work proposed that this domain interacts with the body of the channel and a physical interaction between an N-terminally truncated channel and a soluble PAS domain was demonstrated by FRET. This interaction was sufficient to restore regulation of channel deactivation [12]. Over 30 LQT2 associated missense mutations have been mapped to the amino terminal PAS domain of the HERG 1a subunit [13], [14], [15], [16], [17], [18], [19], [20], [21], eleven of which (K28E, T65P, I31S, F29L, G53R, C66G, L86R, N33T, R56Q, H70R and A78P) have been characterized by electrophysiology[21], [22], [23].

Recent studies have suggested that the underlying cause of LQT2 is the misfolding and retention of the HERG channel in the endoplasmic reticulum (ER) resulting in decreased channel density on the membrane [22], [24]. This has been demonstrated in cell culture to be the case for the majority of missense mutations in the body of the channel and in its carboxy terminus. Studies have also shown that LQT2-associated mutated forms of the HERG 1a subunit are suitable for rescue by temperature or pharmacological chaperones that promote the proper folding of a protein in an active form. For example, the defective trafficking of the N470D mutant is rescued by the addition of the drug E4031 and reduced temperature in cell culture, promoting the idea that increasing the stability of unstable variants can alleviate protein dysfunction [22], [25].

A complete understanding of the molecular properties of LQT2 associated mutations requires not just electrophysiological and cell culture studies but also a biochemical characterization of the impact of the mutations on the folding stability of the protein. In this work, we have focused our attention on nine LQT2-associated mutations distributed throughout the amino terminal PAS domain of the HERG 1a subunit; K28E, F29L, G53R, C66G, L86R, N33T, R56Q, H70R and A78P. We characterized the trafficking properties in cell culture not just in the HERG 1a subunit but also in hetero-tetramers formed by HERG 1a and 1b subunits. Additionally, we measured the thermal and chemicalstability *in vitro* of protein variants with mutations in the PAS domain. Lastly, we explored the ability of the dysfunctional channels to be rescued by temperature and the drug E4031.

## Results

### Trafficking Phenotype of HERG Channels Containing Mutations in the PAS Domain

We characterized the trafficking properties of LQT2 mutant channels in stable cell culture lines expressing either the WT or mutant HERG 1a subunits. We focused our attention on nine LQT2 associated mutations distributed throughout the three dimensional structure of the PAS domain of the HERG 1a subunit; K28E, F29L, G53R, C66G, L86R, N33T, R56Q, H70R, A78P. K28E is a LQT2-mutant that is known to be traffic deficient [21]. The functional characterization of these mutants by electrophysiology has been well described in the literature both in oocytes and HEK293 cells [26], [27]. We also included the mutant E118A. E118A is a non-LQT2 mutation. This mutation is positioned at the tip of the long loop between the 4^th^ and 5^th^ β-strands and it is not expected to alter the structural properties of the domain; moreover, this mutation had been previously shown by electrophysiology not to affect the function of the HERG channel [8].

**Figure 1:**
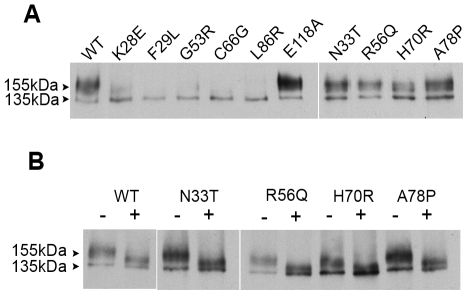
Trafficking phenotype of LQT2 mutant in channels formed by HERG 1a subunit. (A) Western blot of membrane protein extracts from stable HEK293 cell lines expressing full-length WT or LQT2 mutant forms of HERG 1a channel subunit. Mature fully-glycosylated HERG 1a subunit (indicated by 155 kDa label) and immature HERG 1a subunit (indicated by 135 kDa label) are indicated. (B) Western blots of membrane protein extracts from stable HEK293 cell lines expressing full-length WT or LQT2 mutant forms of HERG 1a channel subunit before (−) and after treatment (+) with N-glycosidase F. Western blots were probed with anti-C terminal HERG antibody and were repeated twice (n = 2).

WT HERG 1a subunits show two species on western blot: a band of 155 kDa corresponding to the complex glycosylated form which represents the mature channel form on the cell surface and a lower 135 kDa band, representing the immature core glycosylated form. Figure 1A shows representative western blots of total cell extracts from stable HEK293 cells expressing WT HERG 1a, 9 LQT2 mutant subunits (K28E, F29L, G53R, C66G, L86R, N33T, R56Q, H70R and A78P), and the control mutant E118A protein. The WT and E118A HERG 1a subunits traffic normally as shown by detection of both the mature and immature species. The LQT2 mutants display two different phenotypes: HERG 1a subunits harboring the single point mutants, K28E, F29L, G53R, C66G or L86R show a clear trafficking defect, as only the immature 135 kDa protein band is detected. In contrast, HERG1a subunits harboring the N33T, R56Q, H70R and A78P mutations show both the mature and immature species consistent with a wild-type trafficking phenotype. The steady state level of immature protein in all cases does not appear to change suggesting that these mutations do not affect translation efficiency. To confirm that the two bands detected by Western blot are the result of a different glycosylation level of the protein we treated the samples with N-glycosidase F, an enzyme that removes sugars from asparagine side-chains. This treatment converted the 155 kDa protein bands to a 140 kDa species confirming that the larger band in the blot results from N-glycosylation of the HERG channel (Figure 1B).

**Figure 2:**
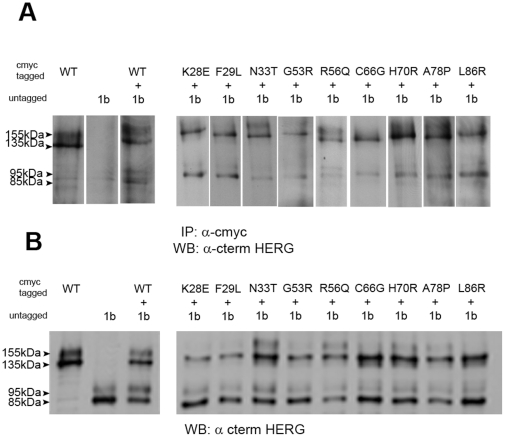
Trafficking effects of LQT2 mutations in hetero-tetrameric HERG channels. (A) Western blot analysis of cell lysates from transiently transfected HEK293 cells co-expressing WT HERG 1a cmyc tagged subunit alone, WT HERG 1b untagged alone, HERG 1a cmyc with HERG 1b or LQT2 mutant HERG 1a cmyc with HERG 1b. Mature forms of HERG 1a cmyc and HERG 1b are indicated by 155 kDa and 95 kDa labels, respectively. Immature forms of HERG 1a cmyc and HERG 1b are indicated by 135 kDa and 85 kDa labels, respectively. Equal amounts of cell lysate were loaded in all lanes as assessed using BCA protein assay quantification Western blots were probed with anti-C terminal HERG antibody and were repeated twice (n = 2). (B) Western blot analysis of immunoprecipitations using anti-cmyc antibody from transiently transfected HEK293 cells co-expressing WT HERG 1a cmyc tagged subunit alone, WT HERG 1b untagged alone , HERG 1a cmyc with HERG 1b or LQT2 mutant HERG 1a cmyc with HERG 1b. Western blots were probed with anti-C terminal HERG antibody and were repeated twice (n = 2).

It has been proposed that *in vivo* HERG channels are hetero-tetramers arising from the assembly of 1a and 1b α-subunits; importantly, HERG 1b does not include a PAS domain. We evaluated the impact of HERG 1a PAS domain point mutations on the trafficking properties of the hetero-tetrameric HERG channel using transient transfection of both proteins in HEK293 cells; this approach allows a simultaneous co-translation of the two HERG subunits. WT HERG 1b subunits show two main species on western blot analysis; a band of 95 kDa representing the complex glycosylated subunit of the mature channel and a lower 85 kDa band, representing the immature core glycosylated form. In order to facilitate our co-expression studies we added a C-terminal myc tag to the WT and LQT2 mutant HERG 1a subunits. We first showed that the LQT2 mutations in HERG 1a cmyc protein are still able to assemble with HERG 1b subunits by carrying out co-immunoprecipitations. Figure 2A shows that immunoprecipitation using an anti-cmyc specific antibody only recognizes the HERG 1a cmyc subunit. On co-expression with HERG 1b we can pull-down the complex formed by both HERG 1a cmyc and HERG 1b proteins. In all cases where we co-expressed LQT2 mutant HERG 1a cmyc subunits with HERG 1b subunits we saw co-immunoprecipitation suggesting that the PAS domain mutations are still able to assemble with the HERG 1b subunit.

Our analysis of lysates from HERG 1a cmyc/HERG 1b co-expressing cells show that the trafficking phenotype (both defective and normal) of the LQT2-associated mutant HERG 1a cmyc subunits defined above was not altered upon co-expression with WT HERG 1b subunit (Figure 2B). The converse effect on HERG 1b, reduction in the levels of mature HERG 1b subunit when co-expressed with trafficking defective HERG 1a mutants, was harder to verify. It has been reported that in heterologous expression systems the level of HERG 1b maturation is only slightly enhanced (up to 3-fold) on co-expression with HERG 1a subunits [28]. Nevertheless, it appears that there is a decrease in HERG 1b maturation when co-expressed with K28E, F29L, G53R, C66G and L86R HERG 1a cmyc subunits relative to experiments where HERG 1b is co-expressed with WT , N33T or H70R HERG 1a cmyc subunits. We could not reproducibly see an effect on HERG 1b when co-expressed with R56Q or A78P HERG 1a cmyc.

### Stability Properties of LQT2-associated PAS Domain Proteins

Our cell culture studies indicate that some of the LQT2 mutations in the HERG PAS domain affect trafficking of the channel, while other mutations do not appear to influence this property. A simple explanation for these effects is that the trafficking defective mutations alter the structure of the PAS domain resulting in improperly folded protein and incompletely processed channel, which does not reach the cell membrane. To explore the effect of the mutations on the structure and stability of the PAS domain in more detail we examined the solution properties of the LQT2 mutations using recombinant PAS domain protein.

The PAS domain from the HERG 1a subunit can be expressed and purified as a stable soluble protein from bacteria, providing an ideal system in which to measure its solution stability properties by Differential Scanning Fluorimetry (DSF). In this method heat induced denaturation exposes the hydrophobic residues normally buried within the core of a protein; binding of the fluorescent dye, Sypro Orange to these hydrophobic residues results in a sigmoidal increase in the fluorescence signal of the dye. The increase in fluorescence is related to the increasing number of protein molecules in the denatured state; the mid-point of the sigmoidal curve is defined as the melting temperature (T_m_) [29].

**Figure 3:**
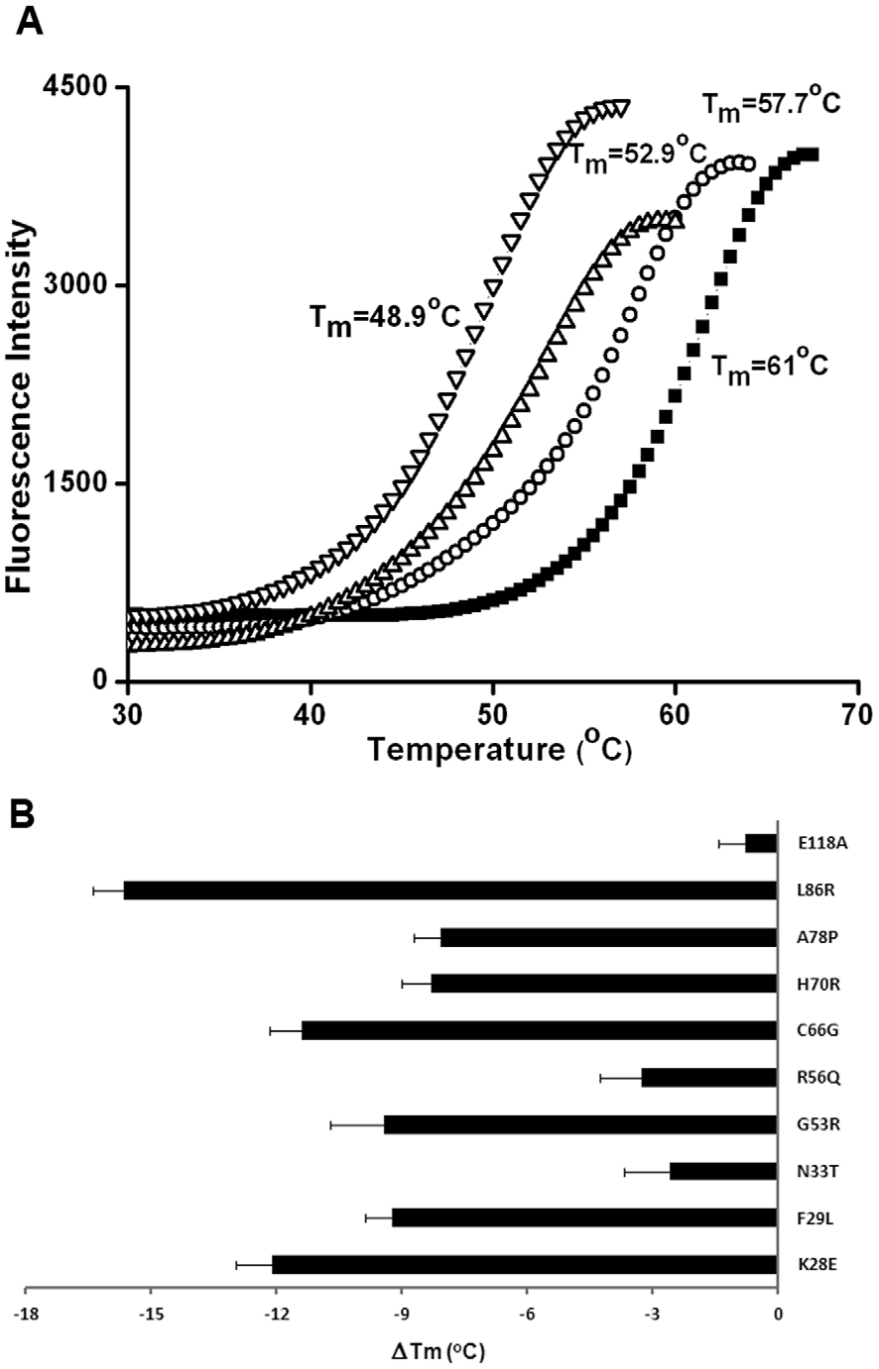
Thermal unfolding of LQT2 PAS domain mutants. (A) Representative melting curves for WT PAS domain (filled squares) and R56Q (open circles), A78P (triangles) and K28E (inverted triangles) PAS domain mutants measured by Sypro Orange fluorescence. (B) Plot of melting temperature changes of mutant PAS domain proteins relative to WT PAS domain measured by Sypro Orange assay. For each protein at least two seperate purifications were analyzed on two seperate days, with 14 replicates per experiment. The Tm was extracted for each curve and averages and standard deviations were calculated for the whole dataset. Standard deviations of the ΔTm were calculated.

All PAS domain proteins analyzed showed a clear single transition curve; representative thermal melting curves and the corresponding T_m_ values are shown in Figure 3A for WT, R56Q, A78P and K28E PAS domain proteins. The ΔT_m_ for each mutant PAS domain compared to the WT domain is shown in Figure 3B and as can been seen not all of the LQT2-associated mutations tested destabilized the PAS domain protein to the same extent. PAS domains containing E118A (control), N33T and R56Q mutations showed melting temperatures (60.2±0.2°C, 58.4±0.6°C and 57.7±0.6°C, respectively) within 3.3 degrees of the WT protein (61.0±0.5°C). These mutations did not alter the trafficking properties of the HERG channel (Figure 1A). However, K28E, F29L, G53R, C66G, H70R, A78P and L86R had much lower melting temperatures; the most extreme being that of the L86R mutant (45.4±0.3°C) followed by K28E (48.9±0.4°C) and C66G (49.6±0.3°C) corresponding to T_m_ values more than 10°C lower than the WT protein. It is clear from these results that mutations that cause trafficking deficiency in the channel are the same that have the most severe effect on the folding stability of the PAS domain in solution. Conversely, mutations that have little impact on the folding properties of the PAS domain do not affect the trafficking properties of the full-length channel.

**Figure 4:**
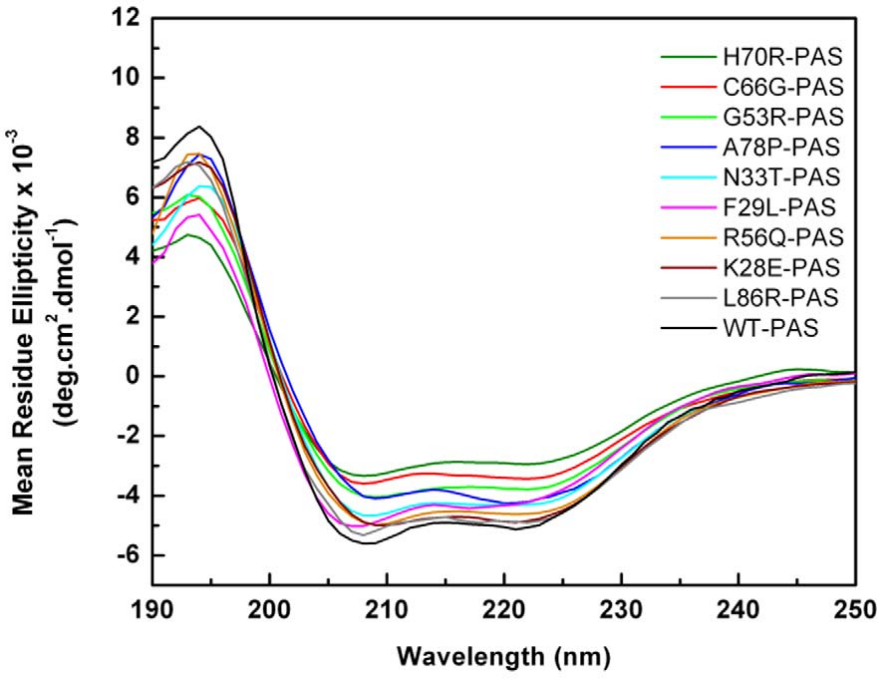
Circular Dichroism spectra. Far-UV CD spectra were recorded between 190 and 250 nm, with 1 nm step resolution. The spectra were averaged over three scans and corrected by subtraction of the buffer signal. Spectra are shown for WT PAS and all nine PAS domain mutants.

Interestingly, the DSF experiments show that the melting temperatures of mutants A78P and H70R (52.9±0.7°C and 52.7±0.2°C, respectively) are only mildly different from F29L and G53R (51.8±0.8°C and 51.6±0.8°C, respectively); however, their trafficking phenotypes are drastically different: A78P and H70R resemble WT channel while F29L and G53R do not give rise to the mature HERG 1a subunit. To further explore the solution properties of the WT PAS domain and LQT2 mutant proteins we used far-UV circular dichroism (CD) (Figure 4). We first evaluated the impact of all mutations on the secondary structure content of the domain as measured by circular dichroism. The α-helical content determined by CD for the WT domain is 23% of the total secondary structure; this value agrees well with the values extracted from the three-dimensional X-ray structure (PDB code: 1BYW). All the mutants analyzed affected the helical content relative to the WT, with reductions between 2 and 6% (Table 1); the mutants that had the most severe effects were G53R, C66G and H70R, which showed an α-helical content between 19% and 17%. The magnitude of the impact in the secondary structure of the domain does not seem to correlate with the trafficking phenotype seen in cell culture since H70R traffics as WT while G53R and C66G do not.

**Table 1:** Estimation of Protein Secondary Structure from CD data.

	% alpha-helix	% beta-sheet	% unordered
WT-PAS	23±0.81	32±0.59	45±0.44
L86R-PAS	23±0.71	32±0.52	45±0.39
K28E-PAS	21±0.74	35±0.77	44±0.61
A78P-PAS	21±0.94	34±0.69	45±0.52
R56Q-PAS	21±0.83	33±0.60	46±0.45
F29L-PAS	20±0.78	34±0.57	46±0.43
N33T-PAS	20±0.82	34±0.60	46±0.45
G53R-PAS	19±0.94	34±0.68	47±0.51
C66G-PAS	19±0.90	35±0.74	46±0.55
H70R-PAS	17±1.00	36±0.74	47±0.56

Far-UV CD spectra were fit to a linear combination of three contributions (alpha-helix, beta-sheet, and unordered structure) using the program CONTIN [32]. Errors result from the fit of the model.

We also measured the folding stability properties by chemical denaturation with urea of the 4 mutants which show similar thermal stabilities but markedly different trafficking phenotypes, (F29L, G53R, H70R and A78P). In these experiments we prepared protein solutions of each PAS variant at increasing urea concentrations and evaluated changes in the circular dichroism spectra; the curves of the CD signal versus urea concentration were then fit to a two state model from which several stability parameters were extracted (Table 2). The WT domain is fairly stable, requiring a high concentration of urea for unfolding (mid-point concentration is ∼6 M) and displaying an unfolding free energy in water of ∼8 kcal/mol. Confirming the thermal unfolding analysis the 4 mutants analyzed by chemical unfolding have lower conformational stability than WT protein. The free energy of unfolding for the mutants is lower, varying between 6.8 and 5.0 kcal/mol, and this is reflected by the lower concentrations of urea necessary for destabilizing the mutant domains, between 4.5 and 4.9 M. However, just like with the melting temperature there is no correlation between the chemical unfolding properties and the trafficking properties of the 4 mutants: the determined unfolding free energies for H70R, F29L and G53R are very similar but the H70R HERG channel traffics well while F29L and G53R do not.

**Table 2:** Protein Conformational Stability Determined from Urea-Induced Unfolding Experiments.

	ΔG(H_2_0)^a^(kcal/mol)	m^b^(kcal/mol/M)	Cm^c^(M)
WT-PAS	8.2±0.4	1.4±0.04	6.3±0.4
A78P-PAS	6.8±0.7	1.5±0.1	4.5±0.6
F29L-PAS	5.0±0.4	1.0±0.1	4.8±0.4
G53R-PAS	5.5±0.9	1.2±0.2	4.6±0.8
H70R-PAS	5.4±0.4	1.1±0.09	4.9±0.3

a ΔG(H_2_0)-free energy change of unfolding in the absence of denaturant.

b m-the dependence of the free energy (ΔG) on the concentration of the denaturant.

c Cm-midpoint of the urea unfolding curve.

The data presented is the result of at least three independent determinations for each protein variant. The errors are calculated from the fit of equation 1 to the experimental curves of CD signal *versus* urea concentration.

Overall these results show that for the mutants, E118A, N33T, R56Q, C66G, L86R and K28E, there is a clear correlation between the magnitude of the changes in the folding stability of the domain and the trafficking properties of the mutant HERG 1a subunits observed in cell culture. This fits well with the idea that a mutation that severely decreases the conformational stability of the PAS domain results in the recognition of the channel by the quality control mechanisms in the endoplasmic reticulum and its subsequent misprocessing. In contrast in the case of F29L, G53R, H70R and A78P, the impact of the mutation on the structure and conformational stability of the PAS domain does not determine the trafficking phenotype of the mutant HERG 1a subunit.

### Rescue Phenotype of HERG Channels Containing Mutations in the PAS Domain

**Figure 5:**
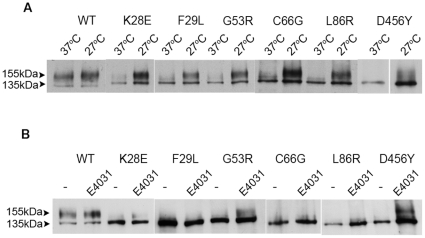
Rescue of trafficking deficient LQT2 mutants. (A) Representative western blot analysis of cell lysate from stable HEK293 cells expressing WT and traffic deficient LQT2 mutations in the HERG 1a subunit grown at 37°C and 27°C. (B) Representative western blot analysis of cell lysates from stable HEK293 cells expressing WT and traffic deficient LQT2 mutations in the HERG 1a subunit grown in the presence (+) or absence (−) of 10 µM E4031 for 36 hours. Western blots were probed with anti-C terminal HERG antibody and were repeated twice (n = 2).

The rescue of the trafficking phenotype can also inform us about the underlying molecular changes induced by the mutations. Importantly, it has been shown that the trafficking deficiency exhibited by HERG 1a subunits with LQT2 mutations in the transmembrane regions can be reversed by incubation at reduced temperature or in a large majority by channel blockers such as E4031 [22]. We therefore asked whether protein processing could also be corrected by temperature or a blocker in the case of PAS mutants. Figure 5A shows representative western blots of cells stably expressing HERG 1a subunits; WT, K28E, F29L, G53R, C66G, L86R or a previously characterized D456Y [22] (a mutation in transmembrane helix S2 which is traffic deficient and is rescued by lower temperature or by addition of blocker) were cultured at 37°C or at 27°C, for either 24 hrs or 48 hrs. In all cases incubation at a decreased temperature results in the appearance of the 155 kDa mature form of the channel showing that the trafficking phenotype has been overcome.

We also evaluated the effect of the channel blocker E4031 (Figure 5B); this small molecule binds with high affinity in the cavity of the ion conduction pathway of the HERG channel [30]. The blocker was added to the cell culture media for 36 hrs. As previously reported the presence of the small molecule rescued the control D456Y mutant since the 155 kDa mature form of the channel is clearly present in the Western blot. In contrast, 4 (K28E, F29L, C66G and L86R) out of the 5 PAS domain LQT2 trafficking mutants tested showed no mature band after the addition of the blocker. Notice that, despite repeated attempts and unlike previously reported in the literature [21], mutant K28E was not rescued by E4031. Incubation of E4031 with cells expressing the trafficking mutant G53R results in the emergence of a higher molecular band.

The rescue experiments show that trafficking defects displayed by all the PAS mutants studied can be rescued by growth at reduced temperature. However, the rescue mechanism by channel blockers that is generally successful for LQT2 mutants positioned in the transmembrane regions was shown to be ineffective with the majority of PAS domain mutants tested.

## Discussion

**Figure 6:**
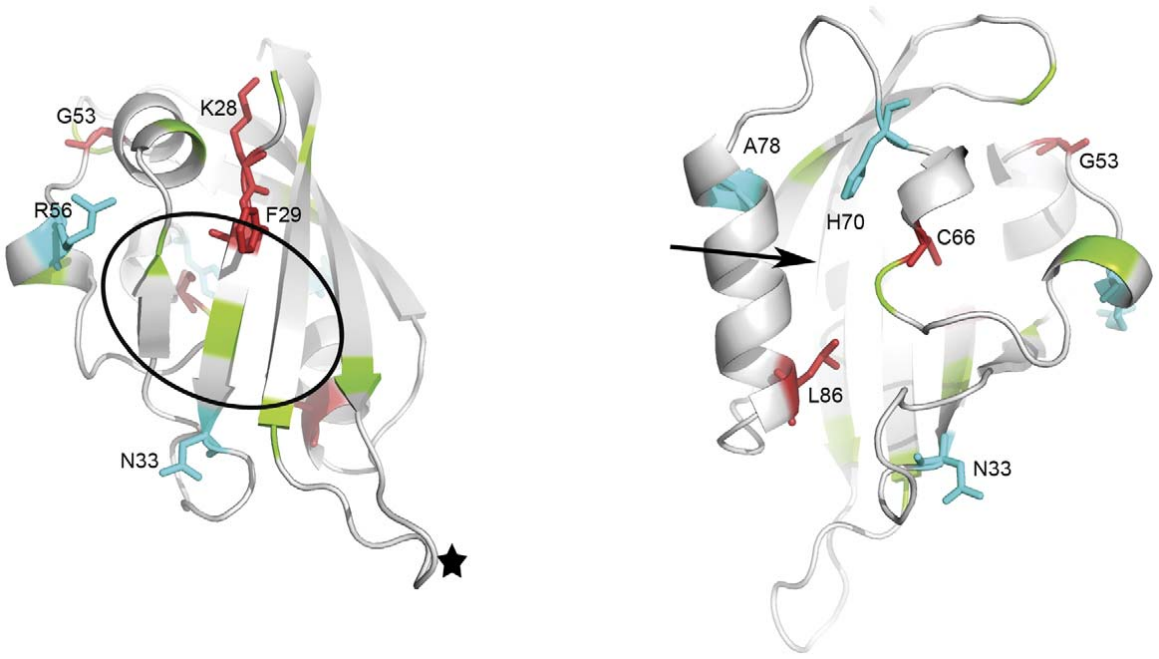
LQT2 mutants mapped onto HERG PAS domain structure. Two views of HERG PAS domain structure X-ray structure (PDB code 1BYW), related by ∼180° vertical rotation. LQT2 mutant positions were mapped: in cyan and stick are mutants included in this study that are not trafficking deficient, in red and stick are the mutants included in this study that are traffic deficient, in green are the position of LQT2 mutants not included in this study. LQT2 mutants in this study are labeled. The black star represents the position of E118, where a control mutation was introduced. The circle roughly indicates the limits of a hydrophobic patch on the surface of domain. The arrow indicates region involved in small molecule binding in PAS domains from other proteins.

LQT2 mutations within the HERG channel are loss of function mutations which reduce I_Kr_ amplitudes and thus prolong cardiac repolarization. The effect of these mutations on the properties of the HERG potassium channel have in general been studied from a functional and cell biology perspective, using electrophysiology and trafficking assays, and have not included biochemical characterizations. As a result there is an incomplete understanding of the molecular changes occurring in the mutant channels. Here we have focused our study on the properties of LQT2 mutants present in the PAS domain of the HERG channel and have coupled an analysis of the trafficking properties with protein stability changes. Importantly, these mutants have been well characterized by electrophysiology both in HEK293 cells [27] and in *Xenopus* oocytes ([26] and are distributed across the three-dimensional structure of the PAS domain (Figure 6).

One of the main points concluded from this work is that the LQT2 mutants in the PAS domain have distinct trafficking properties from the LQT2 mutations in the transmembrane regions. This is clearly reflected in the observation that out of the nine PAS domain mutant channels (K28E, F29L, N33T, G53R, R56Q, C66G, H70R, A78P and L86R) analyzed in this study only five mutants K28E, F29L, G53R, C66G and L86R, show a trafficking defect when expressed in cell culture both in homo-tetrameric channels formed by the HERG 1a subunit alone as well as in hetero-tetrameric HERG 1a cmyc/HERG 1b channels. Our results are consistent with the observations recently reported by Gianulis and Trudeau [27]. In contrast studies of LQT2 mutants in the transmembrane regions have shown that by far the majority of mutants are trafficking deficient [22]. Moreover, the majority of the trafficking deficient mutants tested in our study were not rescued by the pore blocking drug E4031. This is also unlike what has been observed for LQT2 mutants in the transmembrane regions; January and colleagues report that 17 out of the 22 mutants in this region were rescued by E4031 [22]. The differences observed between mutations in the transmembrane region and in the PAS domain most likely reflect the distinct molecular properties between these domains. These two regions form independent folding units since it has been shown that the native channel can be reconstituted by adding the soluble PAS domain to a functional N-terminally truncated channel [12]. Taking this into consideration it is easy to understand that mutations in the PAS domain do not have to have the same effect as mutations in other regions of the channel. It is also easy to understand that binding of a drug to the pore of the channel stabilizes the fold of the membrane buried regions but has no effect on the stability of the cytoplasmic PAS domain. In this context the rescue of trafficking deficient G53R mutant (in the PAS domain) by E4031 is at the moment unexplained. Using the thermal assay we have found that the drug does not directly affect the folding stabilities of this PAS domain mutant in solution (data not shown).

We evaluated the biochemical impact of the mutations by measuring thermal stability of the nine soluble PAS domain mutants and by measuring the chemical stability of a sub-group (F29L, G53R, H70R and A78P). We also estimated the secondary structure content of all domain mutants. Our data shows that mutant PAS proteins K28E, C66G and L86R, which show a defective trafficking phenotype, also have significantly lower folding stability *in vitro*, with melting temperatures more than 10°C lower than the WT PAS domain. This correlation supports the idea that these specific changes in the amino acid sequence cause a destabilization of the PAS domain fold, leading to misprocessing of the channel mutants probably through recognition by the cellular quality control mechanisms in the endoplasmic reticulum. Analysis of the PAS domain structure (Figure 6) suggests reasons for the destabilization of the domain: both Cys66 and Leu86 are part of the hydrophobic core of the PAS domain. C66G would create a cavity and remove many stabilizing van der Waals contacts while L86R introduces the very long and positively charged arginine side-chain in the core, most likely disturbing it. Lys28 is partially buried in a small crevice just before the first β-strand and substitution by a glutamate (K28E) will place the negative charge of the carboxylic group in the crevice possibly disturbing the packing of the β-sheet.

Conversely, the thermal stability of mutants N33T and R56Q is very similar to the WT PAS domain, with melting temperatures that are within ∼3°C of the WT, and the corresponding mutant channels have a normal trafficking phenotype. Moreover, estimation of secondary structure content by Far-UV CD confirms that these mutations have a mild structural impact. Strikingly these mutants have been shown to have the most severe functional effects in electrophysiological studies in oocytes, in particular in the kinetics of channel deactivation and activation, as well as in voltage-dependence of steady-state activation and recovery from inactivation [26]. Electrophysiological studies in HEK293 cells also show severe functional effects in these two mutants [27]. These results support the idea that LQT2 in the case of N33T and R56Q results from direct malfunction of the channel in the membrane. The chemical character of the side-chain substitutions in these two cases (polar for polar) as well as the position of the two mutations (both are exposed on the surface of the PAS domain structure) (Figure 6) justifies the almost lack of change in the conformational stability. Interestingly, both mutants are positioned on the outskirts of a hydrophobic patch on the surface of domain which is thought to mediate the direct interaction with the rest of the channel and it is possible that the mutations affect this interaction. This surface patch was initially revealed by the structure of the PAS domain and its functional importance has been recently reinforced by work on the rescue of LQT2 mutants positioned in this area through co-expression of WT PAS domain [8], [27]. Strikingly many LQT2 mutations in the PAS domain are clustered in or around this patch. In Figure 6 we have mapped many of the described LQT2 associated mutations on the structure of the domain; a view of the domain's β-sheet clearly emphasizes the clustering of mutations in the hydrophobic patch and surrounding areas.

Our results also revealed a group of 4 mutants, H70R, A78P, F29L and G53R, which have similar folding stabilities, evaluated either by thermal or chemical unfolding, but display drastically different trafficking phenotypes. These results suggest that, for these mutations, it is not the destabilization of the fold that determines the trafficking fate of the channel. The mapping of the mutations on the structure reveals that the trafficking deficient mutants are part (F29L) or on the periphery (G53R) of the hydrophobic patch that is supposed to mediate the interaction of the PAS domain with the channel (Figure 6). It is possible that these two mutations affect this interaction leading to a more global destabilization of the channel and misprocessing.

In contrast, H70R and A78P are clustered together on the opposite face of the domain, away from the hydrophobic patch. Curiously, these two mutants are positioned in a region of the domain which is known to form the binding pocket of regulatory small molecules in other PAS domains [31]. Moreover, the functional effects of H70R and A78P are fairly mild relative to other LQT2 mutants in the PAS domain [26], [27]. The position of these two mutants in the domain structure together with their mild functional and trafficking effects raises the possibility that this region participates in an as yet uncharacterized mechanism of the channel.

Overall, this work adds to our understanding of the molecular properties of LQT2-associated mutants. We have directly evaluated the biochemical effect of PAS domain LQT2 mutations on the stability of the domain and have related these results to the trafficking behavior of HERG 1a subunits containing the same mutations expressed in cell culture. This characterization has allowed us to show that there is a diversity of molecular effects associated with LQT2 mutations which most likely have to be considered when developing therapeutic strategies for LQT2 syndrome.

## Materials and Methods

### Plasmids and DNA Constructs

For mammalian cell expression the wild-type (WT) HERG 1a and HERG 1b cDNA's cloned into pcDNA3.1 vector (Invitrogen) were a kind gift from Dr. G. Robertson (University of Wisconsin, Wisconsin). All LQT2 point mutations were generated in the pcDNA3.1 HERG 1a plasmid using standard overlap extension PCR methods and sequence verified. A C-terminal myc-tag was inserted as an FseI/EcoRI fragment downstream of the HERG 1a sequence in pcDNA3.1 and all LQT2 mutants were sub-cloned into this vector and sequence verified. For bacterial cell expression, the corresponding point mutations were introduced by QuikChange site-directed mutagenesis into a previously described construct pGEX4T-PAS [8], encoding the first 135 amino acids of HERG 1a with an N-terminal glutathione S-transferase (GST) tag; mutants were sequence verified.

### Cell Culture and Drug Exposure

HEK293 cells (American Type Culture Collection, ATCC) were grown in Dulbecco's Modified Eagle Medium (DMEM) supplemented with 10% fetal bovine serum, 1% penicillin-streptomysin and Glutamax-1 in the presence of 5% CO_2_. HEK293 cells were transiently transfected in 6-cm culture dishes with 1.5 µg each of WT and LQT2 DNA using calcium phosphate and cell lysates prepared (as outlined below) 48 hrs after transfection. For co-expression experiments HEK293 cells were transiently transfected in 6-cm culture dishes with 1.5 µg each of WT or LQT2 HERG 1a cmyc tagged DNA and untagged WT HERG 1b DNA. Stable cell lines expressing WT or LQT2 HERG 1a subunits were prepared by calcium phosphate transfection of HEK293 cells in 10-cm culture dishes with 5 µg of the appropriate *Ssp* I linearized DNA with subsequent selection and expansion in media containing 600 µg/ml G418. Stable cell lines were grown either at the control temperature of 37°C or at the reduced temperature of 27°C for 24 or 48 hrs. Incubation with HERG channel blocker, E4031 at 10 µM was carried out at 37°C for 36 hrs.

### Cell Lysate Preparation from HEK293 cells for Western Blot Analysis

For cell membrane preparations, similarly confluent 6-cm dishes of cells were washed once with 5 ml of 1× PBS pH 7.5 then scraped into 1.5 ml of ice cold Homogenization Buffer [200 mM NaCl; 33 mM NaF; 10 mM EDTA pH 8.0; 50 mM Hepes pH 7.5; 1 mM PMSF; 1 µg/ml Pepstatin A; 1 µg/ml Leupeptin]. Cells were subjected to 30× strokes of dounce homogenization then spun at 1,500 rpm in a bench top centrifuge at 4°C. The supernatant was removed and spun at 35,000 rpm for 1 hr at 4°C in a Beckman Ti70 rotor. The high speed pellet was resuspended in 100 µl of Buffer [1% NP-40; 150 mM NaCl; 10% glycerol; 5 mM EDTA; 50 mM Hepes pH 7.5; 1 mM PMSF; 1 µg/ml Pepstatin A; 1 µg/ml Leupeptin] and stored at −20°C. For whole cell lysate preparations, similarly confluent 6-cm dishes of cells were washed once with 5 ml of 1× PBS pH 7.5. Whole cell lysates were prepared by resuspending the cell pellet in 100 µl of Lysis Buffer [1% NP-40; 150 mM NaCl; 10% glycerol; 5 mM EDTA; 50 mM Hepes pH 7.5] and stored at −20°C. Both the membrane preparation and cell lysate were mixed with sample buffer such that the final DTT concentration was 100 mM and samples heated at 65°C for 10 min before being subjected to 7.5% SDS-PAGE. Proteins were transferred by semi-dry blotting to a nitrocellulose membrane and incubated overnight at 4°C with a rabbit anti-C terminal HERG antibody (Alomone Labs) and detected by ECL.

### Co-immunoprecipitation

Co-immunoprecipitation analysis was carried out essentially as described, [28] previously; briefly, cell lysates were prepared 48 h post transfection by resuspending cells in solubilization buffer [ 150 mM NaCl, 25 mM Tris-HCl, pH7.4, 20 mM NaEDTA, 10 mM NaEGTA, 5 mM glucose and 0.5% (v/v) TX100, 1 mM PMSF; 1 µg/ml Pepstatin A; 1 µg/ml Leupeptin] followed by incubation on a rotator for 30 mins. at 4°C. Cell lysates were cleared by centrifugation at 10,000×g for 15 mins. at 4°C and quantified using the BCA protein assay (Pierce Thermo Scientific). Cell lysates were precleared with 25 ul of 25% protein G PLUS-agarose slurry (Santa Cruz Biotech.Inc.) for 1 hr at 4°C. Precleared lysates were incubated with 5 ul of anti-cmyc (9E10) monoclonal antibody (Santa Cruz Biotech.Inc.) for 3 hrs at 4°C. Lysates were further incubated with 50 ul of 25% protein G PLUS-agarose slurry for 2 hrs at 4°C. Beads were washed three times with solubilization buffer containing 0.1% TX100 before being eluted in sample buffer containing 200 mM DTT at 65°C for 10 mins and subjected to 7.5% SDS-PAGE and western blot analysis as outlined above.

### Endoglycosidase Analysis

Equal volumes of cell lysate were first denatured in 50 mM sodium phosphate pH 7.5; 0.02% SDS; 10 mM 2-mercaptoethanol at 100°C for 10 min. Triton X100 was added to a final concentration of 1.5% along with 1.25 units of PNGase F (Sigma) and the reaction incubated for 2.5 hrs at 37°C. In control reactions PNGase F was replaced with buffer. Lysates were processed for western blot analysis as described above.

### Protein Expression and Purification from Escherichia coli

Protein purification was as previously described [8]; briefly, the BL21 (DE3) *E.Coli* strain expressing either wild-type (WT) or LQT2 mutant PAS domain clones were grown to 0.9 OD_600_ at 37°C and then induced overnight at 18°C with 1 mM IPTG. Cells were harvested and lysed using a cell cracker in 50 mM Hepes pH 8.0; 150 mM NaCl; 5 mM DTT; 0.1% Tween 20; 1 mM PMSF; 1 µg/ml Leupeptin and 1 µg/ml Pepstatin A. Cell lysate was cleared by centrifugation for 45 min at 17,000 rpm in a Beckman JA25.50 rotor. Cleared lysate was incubated with Glutathione-Sepharose (GE Healthcare) beads overnight at 4°C with gentle agitation in buffer containing 50 mM Hepes pH 8.0; 150 mM NaCl; 10 mM DTT; 5 mM n-octyl-β-D-glucoside; thrombin was added to this slurry to cleave the fusion protein. Unbound cleaved PAS domain protein was further purified by size exclusion chromatography on a Sephadex 200 column equilibrated in buffer containing 50 mM Hepes pH 8.0; 150 mM NaCl; 10 mM DTT. Protein concentration was determined by Bradford assay (Biorad).

### Differential Scanning Fluorimetry (DSF)

Thermal shift assays were performed in the iQ5 Real Time Detection System (Bio-Rad) using 96 well PCR plates. Each reaction well contained 50 µl of 3 µM protein in 50 mM Hepes pH 8.0; 150 mM NaCl; 10 mM DTT and a 2.5× final concentration of Sypro Orange Dye (Sigma S5692 stock solution 5000×). The plate was heated stepwise from 20°C to 85°C with a heating rate of 1°C/min and a hold step of 30 seconds for fluorescence reading every 0.5°C. The fluorescence intensity was measured with excitation at 490 nm and emission at 530 nm. Data were fitted to a Boltzmann equation using OriginPro 7.5 software and the transition temperatures, or melting temperatures, were extracted. For each protein at least two separate experiments, with 14 wells per experiment, were performed. Although the total fluorescence intensity varied the observed standard error of transition temperature (T_m_) was within 0.8°C over replicates.

### Far-UV circular dichroism (CD)

WT and LQT2 PAS domain proteins were expressed and purified from *E. coli* cultures as above except that the size exclusion Sephadex 200 chromatography column was equilibrated with phosphate buffered saline (PBS) pH 8.0; 10 mM DTT. Finally, the protein buffer was exchanged by running on a HiTrap desalting column equilibrated with 100 mM sodium phosphate buffer pH 8.0 and 1 mM TCEP. Protein concentrations ranged from 0.60 mg/ml to 0.80 mg/ml. Circular dichroism experiments were performed on an *OLIS DSM*-2*0* CD spectrophotometer. Far-UV CD spectra were recorded between 190 and 260 nm using a 0.2 mm path length cuvette. CD spectra were run with a step-resolution of 1 nm, an integration time of 5 sec, and using a bandwidth of 0.6 nm. The spectra were averaged over three scans and corrected by subtraction of the buffer signal. The results are expressed as the mean residue ellipticity [è]_MRW_, defined as [è]_MRW_ = è_obs_ (0.1MRW)/(*lc*), where è_obs_ is the observed ellipticity in millidegrees, MRW is the mean residue weight, *c* is the concentration in milligrams per millilitre and *l* is the light path length in centimeters. Protein secondary structure was estimated from the far-UV CD spectra using the program CONTIN [32].

### Chemical Unfolding Experiments

Urea-induced unfolding experiments were performed by dilution of stock solutions of PAS variants to a final concentration of approximately 0.75 mg/ml in the presence of increasing concentrations of denaturant in 100 mM sodium phosphate buffer and 1 mM TCEP pH 8.0. Fresh stock solutions of urea were prepared gravimetrically and their concentration checked by the refractive index. Protein samples with urea were incubated at 25°C for 12 hr to reach equilibrium. The reversibility of the unfolding process was confirmed by circular dichroism spectra after extensive dialysis of urea-denatured samples against 100 mM sodium phosphate buffer, 1 mM TCEP pH 8.0, and compared with the CD spectra of the native samples. The urea-unfolding profiles were constructed plotting the CD signal at 222 nm against urea concentration. Equilibrium unfolding curves were analyzed using a two-state model [Native (N) ↔ Unfolded (U)]. The experimentally observed spectroscopic property (*y*) as a function of urea concentration is the result of contributions from both the native (N) and the unfolded (U) protein populations, and may be directly related to the equilibrium constant and the Gibbs free energy change [Δ*G* (H_2_O)] for the unfolding reaction by Equation 1, according to the linear extrapolation model (LEM) [33]

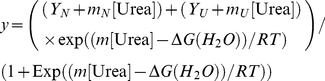
(1)where *Y*_N_ and *m*_N_, *Y*_U_ and *m*_U_ are the intercept and slope of the pre- and post-transition baselines, *R* is the gas constant, and *T* is the absolute temperature. The free energy change in the absence of denaturant [Δ*G* (H_2_O)] and *m*, the dependence of the free energy (Δ*G*) on the concentration of denaturant, were determined by a nonlinear least squares fit of Equation 1 to the unfolding data, using the program Origin (OriginLab Corporation). The concentration of denaturant at the transition midpoint (*Cm*) was also determined for each unfolding profile using Equation 2.
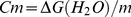
(2)

